# Designing Superoxide-Generating Quantum Dots for Selective Light-Activated Nanotherapy

**DOI:** 10.3389/fchem.2018.00046

**Published:** 2018-03-14

**Authors:** Samuel M. Goodman, Max Levy, Fei-Fei Li, Yuchen Ding, Colleen M. Courtney, Partha P. Chowdhury, Annette Erbse, Anushree Chatterjee, Prashant Nagpal

**Affiliations:** ^1^Chemical and Biological Engineering, University of Colorado Boulder, Boulder, CO, United States; ^2^Renewable and Sustainable Energy Institute, University of Colorado Boulder, Boulder, CO, United States; ^3^Chemistry and Biochemistry, University of Colorado Boulder, Boulder, CO, United States; ^4^Materials Science and Engineering, University of Colorado Boulder, Boulder, CO, United States

**Keywords:** nanotherapeutics, quantum dot, multi-drug resistant bacteria, surface treatment, core-shell nanoparticles, electron paramagnetic resonance (EPR) spectroscopy, electrochemical impedance spectroscopy (EIS)

## Abstract

The rapid emergence of superbugs, or multi-drug resistant (MDR) organisms, has prompted a search for novel antibiotics, beyond traditional small-molecule therapies. Nanotherapeutics are being investigated as alternatives, and recently superoxide-generating quantum dots (QDs) have been shown as important candidates for selective light-activated therapy, while also potentiating existing antibiotics against MDR superbugs. Their therapeutic action is selective, can be tailored by simply changing their quantum-confined conduction-valence band (CB-VB) positions and alignment with different redox half-reactions—and hence their ability to generate specific radical species in biological media. Here, we show the design of superoxide-generating QDs using optimal QD material and size well-matched to superoxide redox potential, charged ligands to modulate their uptake in cells and selective redox interventions, and core/shell structures to improve their stability for therapeutic action. We show that cadmium telluride (CdTe) QDs with conduction band (CB) position at −0.5 V with respect to Normal Hydrogen Electron (NHE) and visible 2.4 eV bandgap generate a large flux of selective superoxide radicals, thereby demonstrating the effective light-activated therapy. Although the positively charged QDs demonstrate large cellular uptake, they bind indiscriminately to cell surfaces and cause non-selective cell death, while negatively charged and zwitterionic QD ligands reduce the uptake and allow selective therapeutic action via interaction with redox species. The stability of designed QDs in biologically-relevant media increases with the formation of core-shell QD structures, but an appropriate design of core-shell structures is needed to minimize any reduction in charge injection efficiency to adsorbed oxygen molecules (to form superoxide) and maintain similar quantitative generation of tailored redox species, as measured using electron paramagnetic resonance (EPR) spectroscopy and electrochemical impedance spectroscopy (EIS). Using these findings, we demonstrate the rational design of QDs as selective therapeutic to kill more than 99% of a priority class I pathogen, thus providing an effective therapy against MDR superbugs.

## Introduction

Antibiotics have been a cornerstone of modern medicine, where molecules like β-lactams are an important and frequently used class. However, the rapid emergence of bacteria that release β-lactamases, and more recently metallo-β-lactamases such as NDM-1, have further proliferated the rise of drug resistance in Enterobacteriaceae, amounting to a major public health concern (Pitout and Laupland, 2008). The increased necessity to address the rise of multi-drug resistance (MDR) in these gram-negative pathogens was highlighted by the first-ever classification of carbapenem-resistant Enterobacteriaceae as a critical class-I pathogen by the World Health Organization (2018). There is an urgent need for the development of novel antibiotics to specifically target the significant increase in MDR bacterial outbreaks, and nanotherapeutics offer an alternative due to their stability, ease of delivery, and facile transport through cell walls. Three different classes of light-activated nanoscale therapeutics have been proposed: metal-nanoparticle induced photothermal therapy (Hirsch et al., 2003; Connor et al., 2005; Huang et al., 2006), small photosensitizing molecules induced photodynamic therapy (Dai et al., 2010; Hamblin and Hasan, 2014), and quantum dot (QD)-based selective-redox (Courtney et al., 2016, 2017; Reynolds et al., 2017), therapies. While non-selective heating of surrounding medium and indiscriminate cell killing by metal nanoparticles reduces their suitability as an antibiotic, lack of tunability or control over the specific reactive oxygen species (ROS) generated by photodynamic therapies, such as non-selective singlet oxygen (Bonnett, 1995; Jarvi et al., 2006; Ma et al., 2008; Gomes et al., 2011; Hamblin and Hasan, 2014), reduces its appeal as a precision therapeutic approach. QDs are colloidal semiconducting nanoparticles with easily tunable optical and electronic properties and have been extensively studied for applications in bio-imaging (Gao et al., 2004; Medintz et al., 2005; Michalet et al., 2005; Gomes et al., 2011). By tuning shape- and size-dependent energies of photoexcited charge carriers in QDs via modulation of their conduction band (CB) and valence band (VB) positions, selective perturbation of redox homeostasis in a cellular environment outlines their selectivity as light-activated therapeutics (Courtney et al., 2016, 2017; Reynolds et al., 2017). More specifically, superoxide-generating QDs have recently been shown as a two-pronged strategy for combating superbugs: first, as therapeutics to selectively eliminate MDR bacteria (Courtney et al., 2016), and second, by potentiating existing pipeline of antibiotics which are nominally ineffective against these potent pathogens (Courtney et al., 2017).

Specific generation of superoxide radical (${\text{O}}_{2}^{•-}$), one of the species characterized as ROS, requires an electron to be donated to an adsorbed oxygen molecules. While several other ROS species such as singlet oxygen and hydroxyl radicals are indiscriminate in their action and reactivity, the impact of superoxide mainly affects critical intracellular targets such as iron-sulfur clusters. Because of limited reactivity of superoxide with cellular targets, superoxide dismutase generated by cells tightly regulates the intracellular redox environment during the normal cellular function. This provides an important opportunity for a precision therapeutic that can selectively generate superoxide at nanomolar doses intracellularly, perhaps controlled by an external stimulus like light, and such exogenous superoxide generation can lead to growth inhibition and cell death (Yu, 1994; Thannickal and Fanburg, 2000; Apel and Hirt, 2004; Valko et al., 2007; Imlay, 2013).

## Results and discussion

### QD material and size

**Figure 1:**
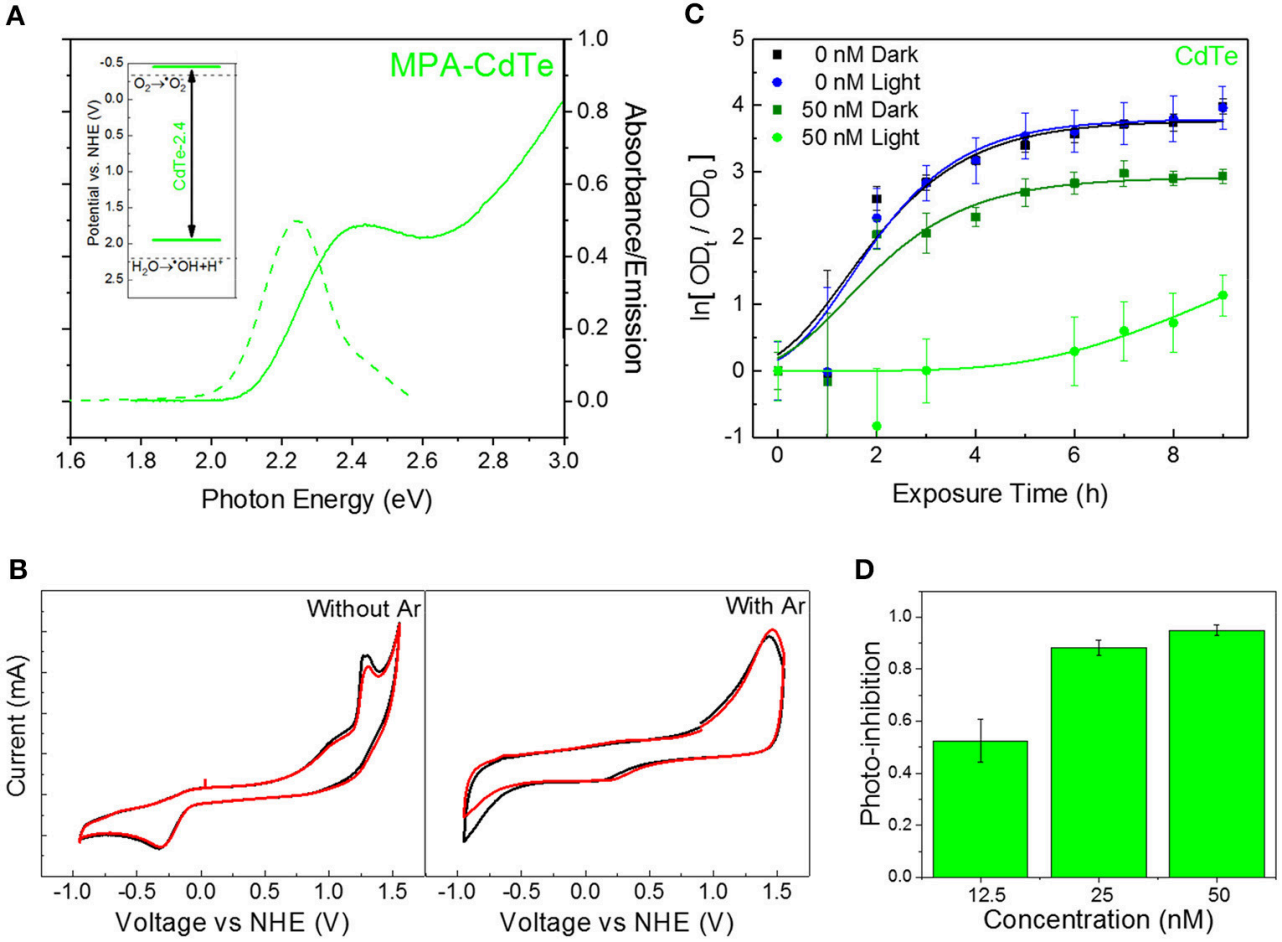
Choice of QD core material and size for superoxide generation. **(A)** Absorbance (solid curve) and PL emission (dashed curve) spectra for CdTe-2.4. Inset shows band positions on NHE scale. **(B)** Cyclic voltammetry measurements for CdTe suspensions in PBS before (left) and after (right) bubbling with argon to remove dissolved oxygen. **(C)** Normalized optical density growth curves of *E. coli* with and without CdTe-2.4 core treatment in light and dark. Solid lines were added by fitting the data to a version of the Gompertz function. **(D)** Photo-inhibition as a function of QD concentration. Data shown in **C,D** is an average of three biological replicates and error bars denote standard deviation.

QDs offer unique advantages for developing this class of redox-selective nanotherapeutics, by offering specific control over redox species generation. Nanoscale material and morphology can be modified to tailor the position of the quantum-confined oxidation and reduction potentials. Furthermore, such a strategy can be effective in developing a tunable class of therapeutics for a wide variety of diseases (Trachootham et al., 2009; Wondrak, 2009). For the specific class of superoxide-generating therapeutic against MDR superbugs, we developed cadmium telluride (CdTe) nanoparticles with a band gap of 2.4 eV (CdTe-2.4)—corresponding to a diameter of 2.7–3.0 nm (Figure 1A). CdTe-2.4 QDs have a CB position at −0.5V on the NHE scale, allowing direct charge injection of photogenerated electrons to adsorbed oxygen on the surface–yielding superoxide radicals (inset, Figure 1A). This was verified by electrochemical cyclic voltammetry studies in the presence and absence of dissolved oxygen (Figure 1B). Furthermore, this 2.4eV bandgap allows the QDs to be strongly activated for therapy by visible light with modest penetration through the human skin (Courtney et al., 2017). We tested photoactivated CdTe-2.4, at concentrations well below the toxic threshold for mammalian cells (Courtney et al., 2016) by tracking the time-dependent optical density (OD) as a measure of bacterial growth. We observed that once exposed to light with energy greater than the nanoparticle bandgap, there is a strong attenuation in the bacterial growth (Figure 1C). This was a clear consequence of light activation since cultures exposed to the same low concentrations of QDs without exposure to light were able to grow well (Figure 1C). To quantify the extent of bacterial growth inhibition with this light-activated treatment, we compared the OD of the treated bacterial culture at a time-point relative to the OD at time *t* = 0 (ΔOD_*x,T*_), with those receiving no treatment (ΔOD_*x,NT*_).

(1) $$\begin{array}{r} I_{P}=1-\frac{\Delta {\text{OD}}_{L,T}}{\Delta {\text{OD}}_{L,NT}} \end{array}$$

This photo-inhibition (*I*_*P*_) increases with higher QD concentrations (Figure 1D) while maintaining the same light intensity, due to stronger light absorption and a larger number of photogenerated intracellular superoxide species. Since the two-fold and four-fold increase in nominal QD dosage increases the intracellular QD concentration and hence superoxide concentration, a similar increase in therapeutic effect can be obtained by improving the uptake of CdTe-2.4 QDs inside cells while keeping the same dosage of QDs, we tuned the surface charge of ligands to modulate cellular uptake.

### Ligand selection

**Figure 2:**
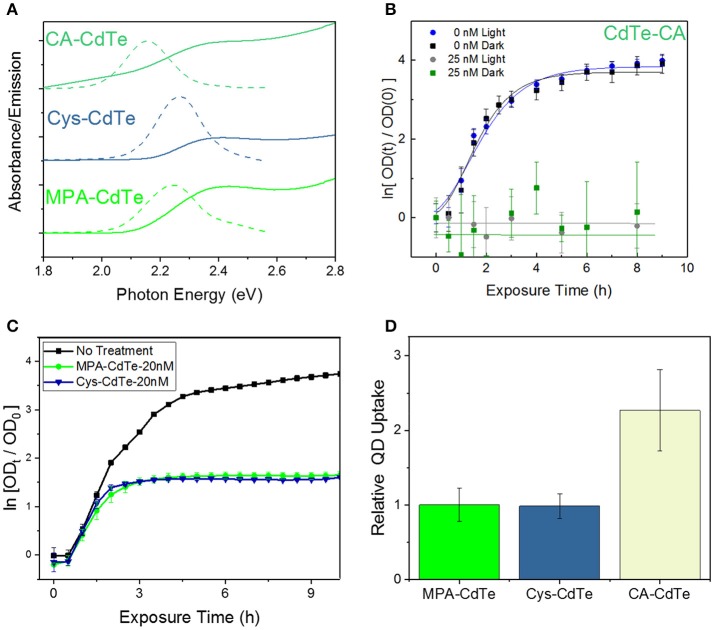
Design of QD ligand and surface charge. **(A)** Optical properties of CdTe QDs coated with MPA, Cys, and CA. **(B)** Optical density (OD) growth curves of *E. coli* normalized to time *t* = 0 exposed to CA-CdTe in light and dark. **(C)** Optical density growth curves of *E. coli* normalized to time *t* = 0 exposed to Cys-CdTe and MPA-CdTe in light. **(D)** Uptake of the MPA, CA, Cys coated QDs into *E. coli*. Data shown in **(B–D)** is an average of three biological replicates and error bars denote standard deviation.

We engineered the QD surface charge by using three different QD ligands with CdTe-2.4 QDs: positively-charged cysteamine ligand-coated QDs (CA-CdTe), negatively-charged mercaptopropionic ligand-coated QDs (MPA-CdTe), and zwitterionic cysteine coated QDs (Cys-CdTe), as shown in Figure 2A and Figure S1. To observe only charge related effects, all ligands had similar sizes and used the same thiol group attachment to the Cd-rich facets on the CdTe QD surface. At the other end, a deprotonated carboxylic acid group and/or amine group provided enough electrostatic repulsion to yield colloidal stability in water. The charge on the surface of nanoparticles can have an important influence on their cellular uptake through the predominantly negatively charged lipid bilayers (especially for MDR gram-negative bacteria studied here) (Lovrić et al., 2005).

To monitor the effect of QD surface charge on cellular uptake and consequent bacterial inhibition, we grew bacterial cultures in the presence of positively-charged CA-CdTe and zwitterionic Cys-CdTe QDs (both with 2.4 eV bandgaps) and compared the results with MPA coated CdTe-2.4. Bacterial growth in the presence of positively-charged QDs (CdTe-CA) exhibit much greater dark toxicity than their negatively-charged and zwitterionic counterparts (Figures 2B,C). While positively-charged CdTe QDs show more than two-fold increase in cellular uptake compared to MPA and cysteine coated QDs (Figure 2D), at concentrations of 12.5 nM, there is no measurable cell growth even after 9 h, when inhibition in both light and dark approach the maximum value of 1. The increased toxicity of using positively-charged ligands has also been reported for other nanoparticles in both Gram-positive and Gram-negative bacteria (Nagy et al., 2012; Feng et al., 2015). This indiscriminate toxicity can be caused due to the strong attachment of the positive-charged QDs to the cellular membrane, negatively charged DNA, RNA, proteins thereby inhibiting key cellular functions leading to cell death. While such indiscriminate cellular killing makes a positively charged ligand unsuitable for selective therapy, the zwitterionic ligands significantly reduce such interactions in biological media, as measured by the hydrodynamic radius, when compared to charged ligands (Choi et al., 2007). The zwitterionic Cys-CdTe showed similar uptake to MPA-CdTe. Zwitterionic ligands have been shown to dramatically increase clearance of QDs from living systems by reducing the hydrodynamic radius (Choi et al., 2007). Taken together with the results showing similar stability and therapeutic effect to MPA-CdTe, we concluded that cysteine would be the optimal choice of ligand for QD design.

### Core-shell QDs

**Figure 3:**
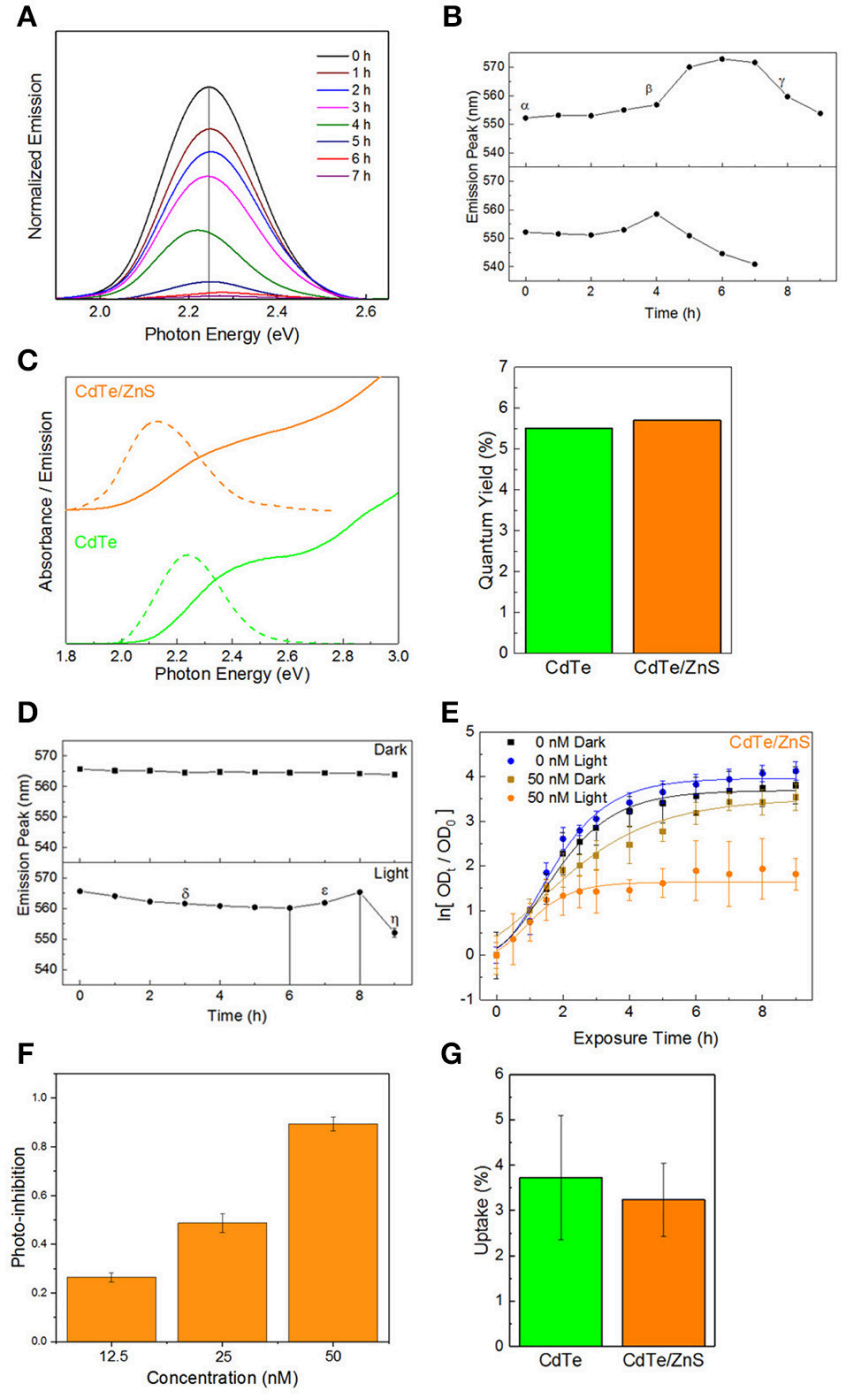
Core-shell QDs for improved stability. **(A)** Normalized emission spectra of QDs over time in PBS during illumination, exhibiting decreased intensity, and shifts in the emission maxima. **(B)** Peak positions as a function of time, exhibiting different regimes of oxidation-instability. **(C)** Optical spectra and quantum yields of the CdTe cores and CdTe/ZnS core-shells. **(D)** Degradation profiles of CdTe/ZnS in PBS with and without illumination. **(E)** Normalized optical density growth curves (with respect to time *t* = 0) of *E. coli* exposed to the core-shells in light and dark. **(F)** Inhibition as a function of QD concentration. **(G)** Uptake of CdTe/ZnS core-shell QDs compared to CdTe cores. Data shown in **(E–G)** is an average of three biological replicates and error bars denote standard deviation.

One of the limitations of the light-activated therapy is the limited length of time that the particles are active (4–6 h) after which surface oxidation leads to vanishingly small therapeutic action (Dumas et al., 2010). To quantify the degradation kinetics, an experiment was conducted where the absorbance and emission spectra of CdTe cores were measured over time in phosphate-buffered saline (PBS) with and without light exposure. Using the photoluminescence spectra (Figure 3A), there is a clear attenuation in signal intensity over time during illumination. Additionally, results show significant shifts in the peak position and an increase in the peak full-width at half maximum—all of which are representative of surface oxidation in a biological media (Schneider et al., 2009). Tracking the position of the emission peak reveals different regimes of particle degradation (Figure 3B). Starting from the initial state (α) there is a regime of general red-shifting (β) which lasts until hours 4 and 6 in light and dark, respectively. This behavior, which has previously been observed *in vitro* (Lu et al., 2008), likely corresponds to the creation of oxygen defects on the nanoparticle surface, which act as lower energy recombination centers. Long duration X-ray photoelectron spectroscopy (XPS) studies of bulk CdTe exposed to aqueous environments report an oxygen-rich surface, consisting primarily of CdO and TeO_2_ (Zeng et al., 2015). After the maximum redshift there is a rapid blue-shifting regime (γ) which indicates that the diameter of the emissive CdTe core is steadily shrinking as CdO and TeO_2_ form. This regime experiences the largest reduction in luminescence intensity. By hour 7, no emission was detectable in the light-exposed sample, indicating that the oxidized shell makes non-radiative recombination the kinetically favored relaxation pathway for photogenerated charges. While the change in photoluminescence intensity may indicate that fewer photoexcited charge carriers are available for charge injection, the main result of the degradation is the shifting of redox potentials and the inclusion of a charge injection barrier against interaction with the external medium. Therefore, we evaluated different surface treatment methods to increase the QD stability while retaining the light-activated antimicrobial action.

A method of increasing the stability of QDs is to use a thin shell of a more stable material around the core. Such core-shell QDs have been used for fluorescent labeling, employing a type-I semiconductor heterostructure between an emissive CdX core enveloped in a thin shell of ZnS (CdX/ZnS) which protects the emission and decreases the toxicity (Kirchner et al., 2005; Chan et al., 2006; Cho et al., 2007). For therapeutic applications, the added stability from thicker shells is offset by an increased tunneling barrier for moving photogenerated charges from the core material to the adsorbate on QD surface (Ipe et al., 2005), thereby reducing the generation of light-activated redox species. There is also the potential effect of different adsorbate-binding affinities between the core and shell material, which can impact the therapeutic effect. Therefore, in order to minimize any negative impacts on the therapeutic action, we investigated CdTe/ZnS core-shell QDs with sub-monolayer thick shells. ZnS shell deposition was identified spectroscopically by characteristic changes in the absorbance spectra and red-shifting emission with increasing shell thickness (Tsay et al., 2004; Figure 3C). Although the shell formation does not have a strong impact on the fluorescence quantum yield (QY) in relation to the cores alone, characterizing the degradation kinetics of the CdTe/ZnS core-shell reveals different regimes of changes and a greater innate stability than the cores (Figure 3D). There is an initial regime of slowly blue-shifting emission (δ). This effect is transient, and a subsequent red-shifting is observed, as seen in the cores (ε), followed by a rapid blue-shift and collapse of the emission intensity (η). These core-shell QDs also show enhanced stability in dark, and the very small blue-shifting observed may be due to the UV light used to test the emission.

Testing the phototherapeutic effect of these stable core-shell CdTe/ZnS QDs reveals a light-activated photo-toxic effect on *E. coli* and a concentration-dependent dark toxicity, in good agreement with previous observations (Figures 3E,F). There is, however, a decrease in the phototherapeutic effect, likely due to the shell material interfering with redox activity of these nanoparticles. This reduction in redox activity is reflected by the growth curves since these changes were not due to different levels of uptake between the cores and core-shell QDs (Figure 3G), despite the core-shell QDs being slightly larger than their uncoated counterparts (Figures S2–S4 in the ESI^†^). This indicates that, for this size regime, the main factor in cell uptake QD surface charge and not QD material. While the CdTe/ZnS core-shell QDs exhibit higher stability in the biological medium, they are less effective as therapeutics due to the reduction in redox activity and perturbation of the electronic structure at the hetero-interface.

### Constituent element overcoat

**Figure 4:**
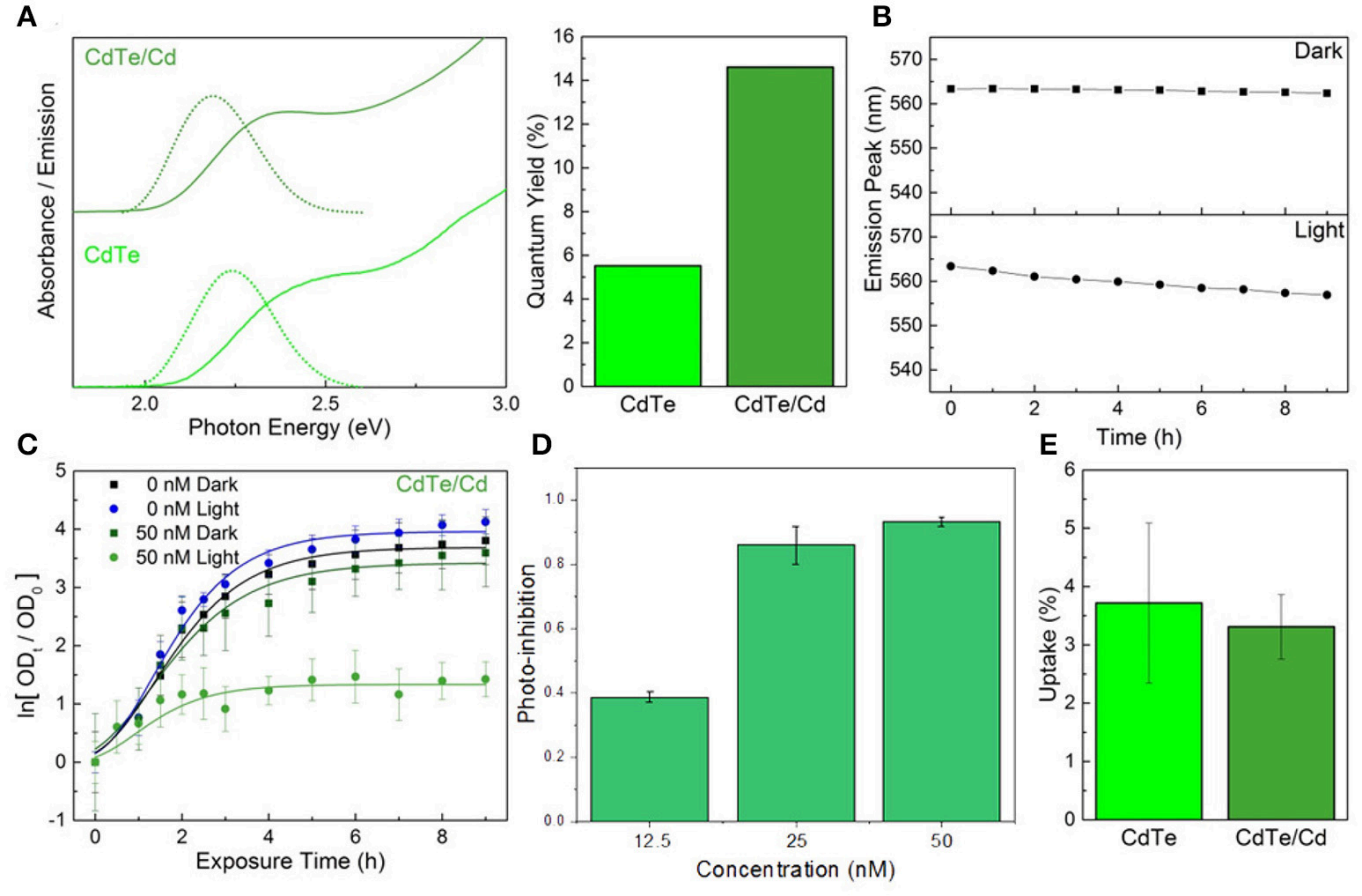
Constituent element overcoat for improved stability and therapeutic effect. **(A)** Optical properties and fluorescence quantum yield of the CdTe/Cd particles compared to cores. **(B)** Emission peak changes during exposure to PBS in light and dark conditions of the core-shells. **(C)** Normalized optical density growth curves of *E. coli* exposed to CdTe/Cd. **(D)** Photo-inhibition as a function of QD concentration. **(E)** Uptake of the CdTe/Cd surface treated QDs compared to CdTe cores into *E. coli*. Data shown in **C–E** is an average of three biological replicates and error bars denote standard deviation.

An alternative to using a core-shell heterojunction for QD passivation is surface treatment by depositing more of the constituent elements on the surface to enhance stability. Depositing excess tellurium, or creating Te-rich facets on the surface, decreases QD stability and degrades the optical properties (Figure S5 in the ESI^†^). However, creating cadmium-rich facets by treatment with excess CdCl_2_ can increase the diameter slightly, thus decreasing the optical bandgap. In addition, the chemical and colloidal stability would likely increase due to the larger number of ligand binding sites (Katari et al., 1994). CdTe/Cd QDs were synthesized by combining CdTe cores (2 μM) with a mixture of CdCl_2_ and MPA in a 1:1 ratio, followed by reacting at 98°C for 15 min. Elemental analysis of these particles indicates there is a 30% increase in cadmium content compared to the cores (0.6 MLE coverage), implying complete passivation of the tellurium facets. The reduction in defects is reflected by the increase in the photoluminescence QY of the cadmium overcoated nanoparticles (Glozman et al., 2001), which is over twice that of the untreated cores, while the slight red-shift in absorbance confirms their larger size (Figure 4A). The cadmium overcoated samples consistently outperformed both the cores and CdTe/ZnS core-shell QDs (Figure 4B). Unlike either, the CdTe/Cd QDs were still luminescent after 24 h of irradiation and underwent much slower rates of degradation during the first 9 h. The degradation curve also consists of the characteristic single monotonic phase of slow blue-shifting. These results also point to a potential application of these CdTe/Cd QDs for optical tracking and bio-imaging due to their apparent high QY and stability in biological media. As there are no exposed tellurium-rich facets to readily oxidize, degradation only involves CdO buildup on the surface followed by slow inward diffusion. In addition, minimal QD aggregation was observed during emission measurements, indicative of an increase in the number of bound capping ligands on the surface which predicts superior colloidal stability. When tested *in vitro*, the CdTe/Cd QDs induced a stronger photo-toxic effect than the CdTe/ZnS core-shell QDs, while the inherent-toxicity remained consistent within the measured concentration range (Figures 4C,D). As with the CdTe/ZnS core-shell QDs, there was also no observed difference in uptake between the CdTe/Cd and CdTe core nanoparticles (Figure 4E).

### Spectroscopic evaluation of QD design

#### Electrochemistry

**Figure 5:**
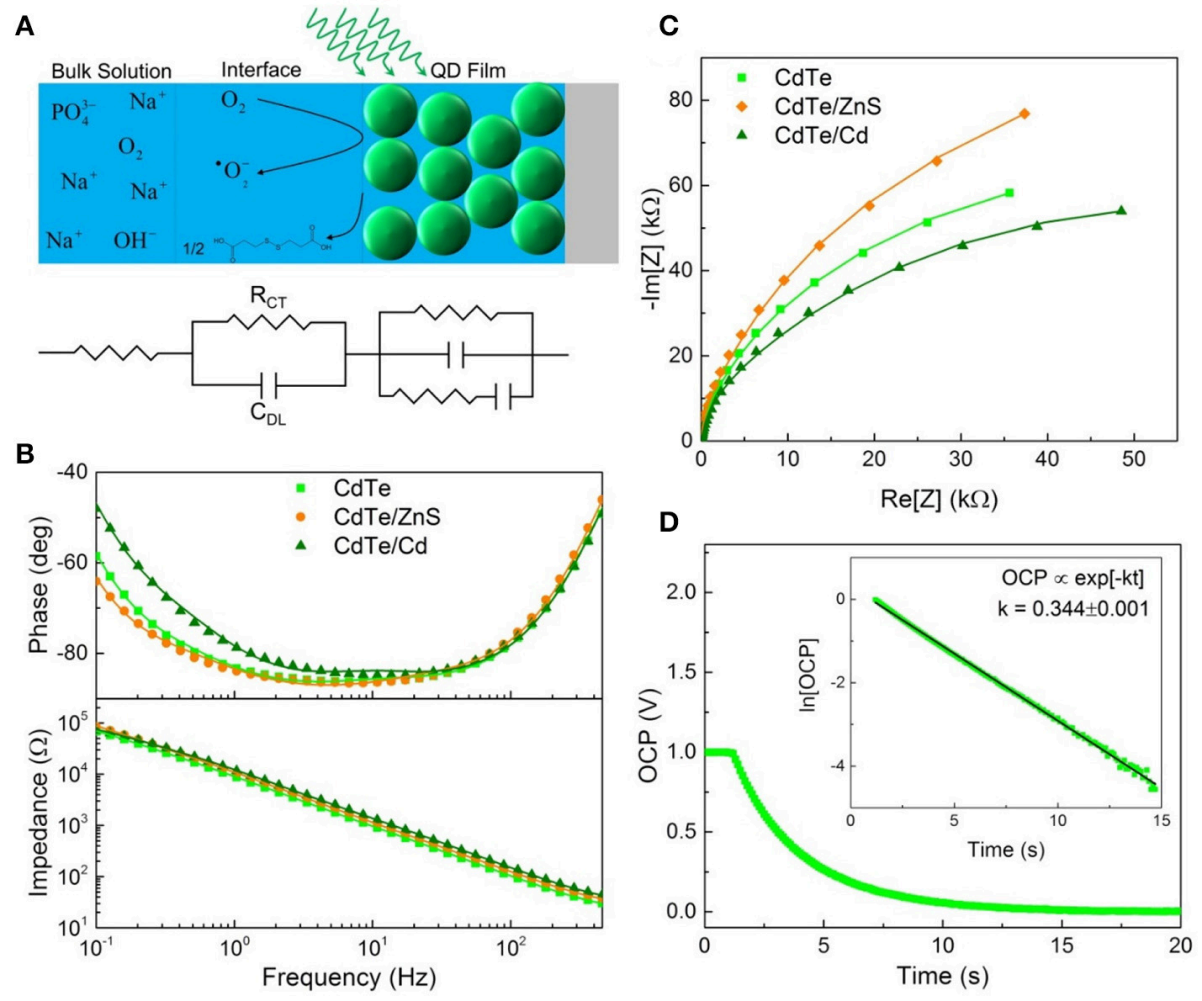
Electrochemical characterization of QD design. **(A)** Schematic illustrating EIS measurement and equivalent circuit components for each part. **(B)** Bode and **(C)** Nyquist plots used to determine the interfacial parameters reported in Table 1. **(D)** Open circuit potential decay of the cores with the linearized plot used to extract the rate of charge injection inset.

Following light-activation, photogenerated charges in the QDs are transported to the surface near sites of adsorbed chemical species and are subsequently injected into the adsorbate which forms the products of the photochemical process (Figure 5A). The kinetics of these photoredox processes and the resistance to each step can be characterized using electrochemical impedance spectroscopy (EIS). Using analysis of Bode and Nyquist plot, and an equivalent circuit diagram to obtain the solution resistance, resistance to charge injection to adsorbate (R_CT_), capacitance of the electrical double layer (C_DL_), and inherent resistance to charge transport and defect state density for the semiconductor QDs (Figures 5B,C). Comparing resistance to charge injection between CdTe core, CdTe/ZnS core-shell, and CdTe/Cd overcoated QDs, we observed that CdTe core QDs have the lowest resistance, as expected. CdTe/ZnS and CdTe/Cd were found to have 5- and 8-fold higher resistance to charge injection (Table 1). Also, while the double layer capacitance of CdTe cores and core-shell QDs was similar, the Cd overcoated QDs had lower capacitance, indicating easier transport of redox species even though the interfacial charge injection resistance is higher. This can likely be explained by a higher adsorption affinity of the chemical species (oxygen) on the cadmium-rich surface. Further analysis of trapped charges and transport within the semiconductor QD did not reveal any appreciable differences.

**Table 1:** Charge injection rate for photogenerated electrons (k), interfacial resistance to charge injection (R_CT_), and capacitance of double layer (due to charge trapping at the interface, C_DL_), measured using electrochemical impedance spectroscopy.

	**CdTe**	**CdTe/ZnS**	**CdTe/Cd**
k (ms^−1^)	0.344 ± 0.001	0.317 ± 0.002	0.31 ± 0.01
			0.92 ± 0.05
R_CT_ (kΩ·cm^2^)	1.02 ± 0.02	5.03 ± 0.01	8.74 ± 0.03
C_DL_ (μF·cm^−2^)	73.4 ± 0.4	63.5 ± 0.2	35.0 ± 0.1

**Figure 6:**
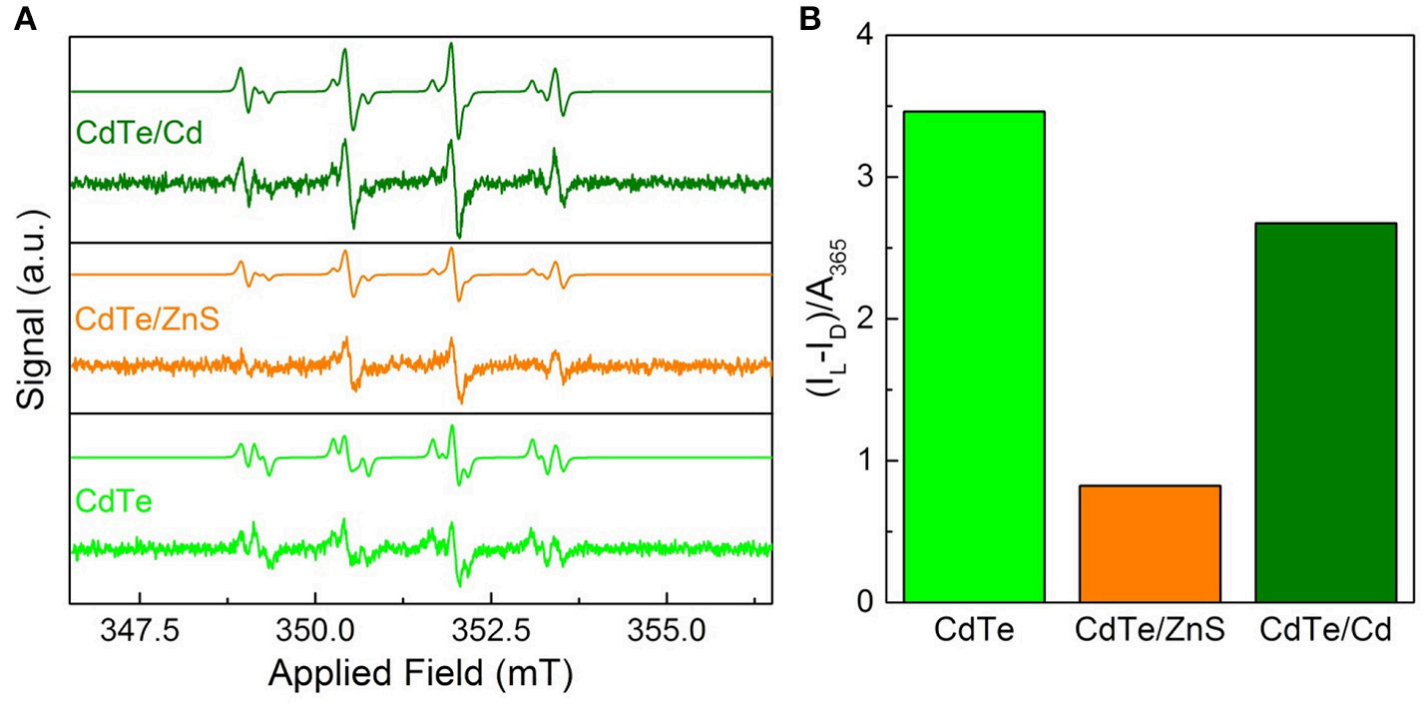
Superoxide radical characterization via EPR for optimal QD design. **(A)** EPR spectra of the cores and core-shells before after 30 s of 365 nm irradiation with simulations based on a combination of hydroxyl- and superoxide-DMPO adducts. The dismutation of superoxide radical generated by QDs leads to hydroxyl radical during EPR measurements (Courtney et al., 2017). **(B)** Integrated radical signal after dark subtraction (I_L_–I_D_) and normalization by the sample absorbance at the exciting wavelength.

To further probe the interfacial redox kinetics for therapeutic interventions, we also tracked the open circuit potential (OCP) decay in the respective QDs as a function of time (Figure 5D). The kinetics of OCP decay shows the overall photochemical process is faster in CdTe core QDs compared to the CdTe/ZnS core-shell QDs (Figure 5C). This observation is consistent with the quantified ROS radical generation observed via quantitative measurements of these radical species via Electron Paramagnetic Resonance Spectroscopy (EPR, Figure 6) and phototherapeutic effect *in vitro*. In CdTe/Cd overcoated dots, we observed two modes of decay: one is the faster recombination between photogenerated charge carriers, and the second is the charge injection and photochemical generation of radical species. This observation explains the anomaly between higher QY and depressed ROS generation observed by EPR. While CdTe/Cd overcoated QDs improve the QY and have higher number of photogenerated charges for ROS radical formation, the increased resistance to charge injection leads to recombination (radiative and non-radiative) before they can be injected into the adsorbed oxygen to form superoxide radicals. Therefore, these measurements provide detailed insights and important design rules for making optimizing LARS production.

#### EPR spectroscopy

To probe the efficacy of phototherapeutic action in both cores and surface treated QDs (CdTe/ZnS core-shell QDs and CdTe/Cd overcoated QDs), we tested them using EPR spectroscopy. Previous studies have shown the formation of radicals upon light stimulation of nanoparticles (Ipe et al., 2005; Gao et al., 2014). These measurements were performed with the goal of identifying any short-lived radical species that are formed upon illumination using 5,5-dimethyl-pyrroline-N-oxide (DMPO) as a long-lived spin trap. The spectra of samples exposed to light show two components corresponding to DMPO adducts formed from hydroxyl and superoxide radicals (Figure 6A) (Finkelstein et al., 1980). This was confirmed by simulating the spectra as a combination of the two adducts using hyperfine coupling constants of a_N_ = 14.90 G and a_H_β = 14.93 G for DMPO-OH and a_N_ = 14.2 G, a_H_β = 11.4 G, and a_H_γ1 = 1.2 G for DMPO-OOH in good agreement with established characteristic hyperfine constants (Buettner, 1987). The presence of hydroxyl and superoxide radicals indicate ROS as the toxic mechanism. These features are consistent for the cores and both of the core-shell particles, indicating that neither the interfacial states in CdTe/ZnS nor the increased size of the CdTe/Cd alters the electronic structure sufficiently to change the mechanism. In dark, there was a minor signal originating from the DMPO-hydroxyl radical adduct in each case, likely due to ambient light stimulation during sample preparation, during which completely light-free conditions were not possible. To further quantify the phototherapeutic effect observed, we measured the relative free-radical production by each QD by integrating the EPR spectra and normalizing to the QD absorbance (Figure 6B). Our experiments clearly show that CdTe core QDs have significantly higher yield of ROS species compared to CdTe/ZnS core-shell QDs, which explains the observed difference in their respective phototherapy. The production from CdTe/Cd shows attenuation to a lesser degree. Together, these measurements provide valuable design rules for QD surface treatments towards nanotherapeutic applications, we conducted EIS investigations of the kinetics of redox generation in core and surface treated CdTe QDs.

### *In vitro* testing of QD design

**Figure 7:**
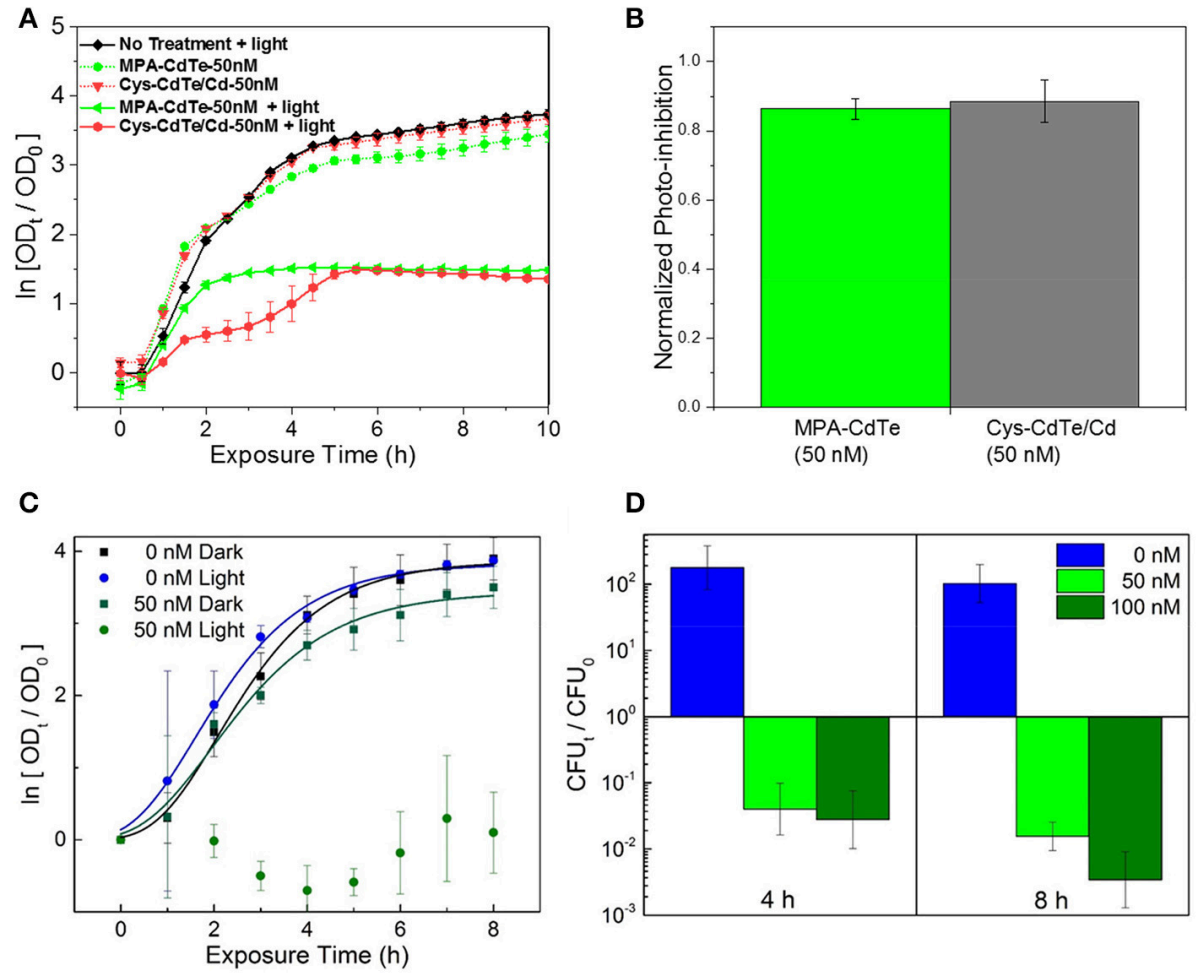
Efficacy of optimal QD design in *in vitro* cultures. **(A)** Normalized optical density growth curves (with respect to time *t* = 0) of *E. coli* exposed to Cys-CdTe/Cd and MPA-CdTe. **(B)** Growth inhibition from rationally designed Cys-CdTe/Cd compared to MPA-CdTe. **(C)** Optical density growth curves of NDM-1 expressing *K. pneumoniae* exposed to CdTe/Cd in light and dark. **(D)** Colony forming unit (CFU) analysis of NDM-1 expressing *K. pneumoniae* cultures at 4 and 8 h of light exposure with respect to CFU at time *t* = 0. Data in **A–D** represents average of three biological replicates and error bars denote standard deviation.

We evaluated the therapeutic effect of optimal QD design, especially ligand selection and surface treatment, by *in vitro* testing and comparison between Cys-CdTe/Cd when compared to untreated MPA-CdTe (Figures 7A,B). In cell culture with light exposure, we found that desired phototoxic effect was consistent between the Cys-CdTe/Cd and MPA-CdTe. While phototoxicity of Cys-CdTe/Cd was similar to uncoated MPA-CdTe, their increased stability provides sustained therapeutic effect against pathogens. We also investigated the therapeutic efficacy of these stability-enhanced CdTe/Cd nanoparticles QDs in countering a WHO priority I MDR Enterobacteriaceae *Klebsiella pneumoniae* expressing New Delhi metallo-beta-lactamase 1 (NDM-1), an enzyme that offers resistance to a broad range of beta-lactam antibiotics including last-resort antibiotics such as carbapenems. This highlights the pandemic problem of drug-resistance and the need for novel antibiotics, and it served as an important test for our nanotherapy. Low concentrations (50 nM) of CdTe/Cd nanoparticles were added to the growth media with these isolates, and the MDR strain grew well in dark (Figure 7C). Nevertheless, on light-activation, no growth was observed in these strains when measuring OD. To further probe the bactericidal effect of the CdTe/Cd nanotherapeutic using colony forming unit (CFU) analysis. Our results show that while untreated strains grew more than 100-fold in 4–8 h, we saw > 90% reduction in 4 h and >99% killing of *K. pneumoniae* at 8 h, compared to the initial bacterial population at *t* = 0 h (Figure 7D). This highlights the efficacy of our nanotherapeutic against potent MDR clinical strains. Taken together, the stability, photo-toxicity, and uptake measurements clearly indicate that a Cd-overcoat is a better choice for the nanotherapeutic QDs than a ZnS shell or no surface treatment.

## Conclusion

In conclusion, we have demonstrated the design of an effective QD-based selective-redox therapeutic, as well as the effect of different surface treatments on this QD light-activated nanotherapy. We showed that while different QD materials demonstrate different biocompatibility, the cellular uptake is primarily governed by the charge on the QD surface. Positively charged CA-coated CdTe QDs showed higher uptake by the cells compared with the negatively charged (MPA) and zwitterionic (Cys) CdTe QDs. While CdTe/ZnS core-shell QDs improve the chemical and colloidal stability of QDs in biologically-relevant media, the enhanced resistance to charge injection for photoredox processes and changes in the interfacial electronic states makes them less attractive as candidates for antimicrobial applications. Using cationic over-coating with CdTe/Cd QDs we demonstrate higher stability and maintain phototherapeutic effect as the CdTe cores, makings them better candidates for those applications. We also tested these CdTe/Cd as therapeutic against a clinical isolate of *K. pneumoniae* expressing NDM-1, which was resistant to β-lactams including carbapenem, and found that it killed more than 99% of the initial bacterial population in 8 h, indicating the potential for application of this nanotherapeutic. Finally, we showed that Cys-coated CdTe/Cd QDs offer improved chemical stability without inhibiting photo-toxicity. Therefore, this study provides detailed physical insights into the mechanism and kinetics of QD nanotherapeutics and provides important design rules for utilizing surface treatments to improve the stability and selective redox interventions using colloidal QDs.

## Methods

### Core synthesis

CdTe cores were synthesized using the materials and methods described in our previous publication (Courtney et al., 2016). Briefly, a reaction mixture was made by mixing a stock solution of degassed CdCl_2_ (0.4 mg/mL; Sigma) and MPA (1 mg/mL; Fischer) in DDW with additional degassed DDW in equal volumes (e.g., 500 μL Cd solution, 500 μL DDW), 0.5 M NaOH (volume: 2% of total), and a solution of NaHTe (40 mg/mL, volume: 0.2% of total). The reaction mixture was then split into 100 μL aliquots and heated to 98°C for 1.5 h in a thermocycler. The resulting QDs are sterile if autoclaved materials are used throughout. Before use in plate assays the QDs were first bulk centrifuged at 15 krpm for 3 min to pellet unreacted starting material and poorly-passivated dots, and 200 μL of the QD stock was then filtered and washed twice with 100 μL of sterile pH 11 water in an ethanol sterilized 4K Nanosep filter (Pall)—5 min at 10 krpm for each filtration and wash step, residual ethanol was removed prior by rinsing the filter with 100 μL pH 11 water. After the final wash, the dots were re-dispersed in pH 11 water and the concentration was determined using optical correlations (Yu et al., 2003). QDs were mixed with the relevant medium before integration with cells.

### Cysteamine ligand exchange

A stock of cysteamine-hydrochloride (CA; Sigma) was created by dissolving CA (7.7 mg, 0.10 mmol) in 1 mL of pH 6 water. This was used to re-disperse CdTe-2.4 cores which were filtered and washed twice with double-distilled water (DDW). The QDs were then kept in the dark at room temperature overnight. Prior to use the particles were bulk-centrifuged at 10 krpm for 5 min to remove poorly-passivated QDs and washed in a similar manner using PBS (Lovrić et al., 2005).

### ZnS core-shell synthesis

A stock 100X solution of zinc and sulfur sources was created by dissolving Zn(NO_3_)_2_·6H_2_O (609 mg, 5.57 mmol; Sigma) and thiourea (75 mg, 1.0 mmol; Sigma) in 10 mL ddH_2_O. For a synthesis, 100 μL of the 100X stock was diluted into 10 mL of freshly de-gassed ddH_2_O which served as the zinc-sulfur precursor stock. 200 μL of CdTe-2.4 stock was filtered, washed twice and re-dispersed with pH 11 water. This solution was then diluted to 2 μM. The reaction solution consisted of the filtered QDs and the precursor stock in a 1:1 ratio, with 10 μL of 0.5 M NaOH per 500 μL of reaction volume. This mixture was then divided into 100 μL PCR tubes and reacted at 98°C for 1 h. Prior to use in cell cultures, they were filtered and washed as previously described. Due to changes in the optical properties the absorbance after synthesis was used to calculate the extinction coefficient at 400 nm for the 1 μM stock, which was then used to calculate the concentrations post-filtering.

### Cd-overcoat synthesis

A Cd-MPA stock was prepared and degassed as previously described for the core syntheses. 200 μL of CdTe-2.4 stock was filtered, washed twice, re-dispersed with pH 11 water, and then diluted to 2 μM. The QD and Cd-MPA stocks were mixed in equal volumes with 10 μL of 0.5 M NaOH per 500 μL of reaction volume. The reaction solution was then divided into 100 μL PCR tubes and reacted at 98°C for 15 min.

### Synthesis chemicals

3-Mercaptopropionic acid (≥99%) was purchased from Acros Organics. Cadmium(II) chloride (technical grade), ammonium fluoride (≥98%), and hexamethylenetetramine (≥99.0%) were purchased from Sigma Aldrich. Tellurium −325 mesh powder (99.99% metal basis) was purchased from Alfa Aesar. Sodium borohydride (98%), sodium hydroxide (≥97.0%), and ethylene glycol (certified) were purchased from Fisher Scientific. Compressed nitrogen (prepurified) and oxygen (ultrahigh purity) were purchased from Airgas. Ethanol (200 proof) was purchased from Decon Laboratories INC.

### Culture conditions

Colonies of *E. coli* (MG1655) were grown on solid Luria Bertani (LB, Sigma Aldrich)-agar media overnight at 37°C from freezer stocks (40% glycerol, −80°C) and stored at 4°C. For a microplate assay, three individual colonies were grown overnight in LB and diluted 1:100 when incorporated with the various QDs in fresh media. Separate 96-well flat-bottom plates were prepared for light and dark conditions, the OD of which was measured using a Tecan GENios at 562 nm. Plates were shaken at 225 rpm in a 37°C incubator between measurements. The dark plate was wrapped in aluminum foil while the edge of the light plate was sealed with parafilm to reduce evaporation. The light source was modulated before each experiment to provide the desired intensity and was equipped with a 400 nm longpass filter (ThorLabs FGL400) and a 350-700 nm bandpass filter (FGS900-A) to remove UV and IR light. The fits were done using Gompertz function, with growth rate (μ), stationary phase population (*S*) and λ is the lag time: (Zwietering et al., 1990).

(2) $$\begin{array}{r} \ln\;\left(\frac{{\text{OD}}_{t}}{{\text{OD}}_{0}}\right)=S\cdot \exp\left\{-\exp\left[\frac{\mu \cdot \exp\left(1\right)}{S}\left(\lambda -t\right)+1\right]\right\} \end{array}$$

### Nanoparticle characterization

All absorbance measurements were obtained with a VWR UV-1600PC spectrometer at 1 nm resolution. Quantum yields (QY) were determined via comparison with a fluorescein isothiocyanate (FITC, Sigma) standard using a NIST calibrated Photon Technologies International fluorimeter for each sample, with emission measured from 485 to 800 nm using 475 nm excitation. Each QY was calculated using Equation (3) (Grabolle et al., 2009). High-resolution transmission electron micrographs (Figures S4, S5) were obtained in the CAMCOR facility at the University of Oregon, and with a Philips CM 100 at 80 kV. Cores exhibited an average diameter of 2.9 ± 0.3 nm, with the core-shells being slightly larger with CdTe/ZnS and CdTe/Cd averaging 3.2 ± 0.5 and 3.1 ± 0.5 nm, which matches the ICP observations of single monolayer-regime coverage.

(3) $$\begin{array}{r} \frac{\Phi_{QD}}{\Phi_{FITC}}=\frac{A_{FITC}\int_{485}^{800}I_{QD}\lambda d\lambda }{A_{QD}\int_{485}^{800}I_{FITC}\lambda d\lambda } \end{array}$$

### Degradation studies

QDs were centrifuged and filtered in the same manner used to prepare stocks for biological assays. Two samples of each type were prepared in PBS to simulate a biologically relevant medium. One was kept in dark, while the other was illuminated using the same light intensity as the assays. Emission spectra were recorded using 365 nm excitation and a calibrated Ocean Optics USB4000 detector.

### Uptake studies

Three cultures were grown overnight and diluted 1:10 into PBS with the QDs at 100 nM total concentration. The cultures were then shaken for 1 h at 37°C and collected into centrifuge tubes. The tubes were spun at 15 krpm at 3 min and the supernatant was removed. The cell pellet was then washed twice with PBS and once with DDW using this procedure. The pellet was then dispersed in ~300 μL DDW for storage (final volume was recorded after dispersion).

ICP-MS samples were prepared by diluting 25 μL of the samples to 1 mL total volume. Standards were prepared within the limits of the possible concentration range for comparison. This analysis provided the raw element composition of the samples, which was used to calculate the signal corresponding to specific concentrations. The monolayers-equivalent addition of Cd and Zn for the CdTe/ZnS and CdTe/Cd samples was calculated by translating the signal to the number of atoms in the core initially (calculated based on the measured core diameter using TEM) and adding the overcoated atoms to the surface and calculating that effective area relative to the initial surface area of the particles. The percentage uptake reported in the figure are defined using a mass balance comparing the total number of particles associated with the cells with the initial number introduced into the cultures.

### EPR spectroscopy

Nanoparticle samples were filtered and washed three times with pH 11 water and once with DDW before DMPO (Dojindo) was added (1 vol%), and were then kept in dark conditions as much as possible prior to measuring. Spectra were obtained on a Bruker Elexsys E 500 spectrometer with an SHQE cavity at 7 mW microwave intensity. Samples were loaded into four quartz capillaries for measurement. Samples were first measured in dark to provide a baseline signal, then were illuminated with 365 nm light for 30 s and immediately re-measured. Quantification of the radical products was accomplished by fitting the spectra to a sum of the theoretical spectra using the following hyperfine coupling constants to simulate each adduct. DMPO-OH: a_N_ = 14.90 G, a_H_β = 14.93 G; DMPO-OOH: a_N_ = 14.2 G, a_H_β = 11.4 G, a_H_γ1 = 1.2 G. Quantification was done by calculating the double integral of the fit spectrum and normalizing the resulting area by the sample absorbance at 365 nm.

### EIS

Electrochemical Impedance Spectroscopy (EIS) was conducted using a Bio-logic SP-200 potentiostat/galvanostat. Using a three-electrode (working electrode: CdTe NPs drop-casted thin film, counter electrode: platinum wire, reference electrode: Ag/AgCl) configuration, the experiments were conducted in a frequency range from 100 kHz to 100 mHz, with an amplitude of 10 mV AC polarization on open-circuit potential (OCP) vs. reference electrode. Bode plot and Nyquist plot were extracted from the measurements and fitted to the suggested equivalent circuit using the Z-fit function in EC-lab software (Bio-logic). Open-circuit potential (OCP) decay/relaxation was conducted using the same three-electrode setup to evaluate the charge transfer characteristics on the QD-electrolyte surface. The measurements were first taken in the dark until stable open-circuit potential was obtained followed by irradiating it with a 300 W xenon lamp. The irradiation was turned off once stable open-circuit potential was reached. The OCP decay curve was recorded until the OCP no longer changed.

## Author contributions

SG, ML, F-FL, YD, CC, PC, and AE: conducted the experiments; PN: designed the experiments; PN and AC: supervised the project; PN, SG, and ML: wrote the manuscript, with input from all authors.

### Conflict of interest statement

The authors declare that the research was conducted in the absence of any commercial or financial relationships that could be construed as a potential conflict of interest.

## References

[B1] Apel K. Hirt H. (2004). Reactive oxygen species: metabolism, oxidative stress, and signal transduction. Annu. Rev. Plant Biol. 55, 373–399. 10.1146/annurev.arplant.55.031903.141701 15377225

[B2] Bonnett R. (1995). Photosensitizers of the porphyrin and phthalocyanine series for photodynamic therapy. Chem. Soc. Rev. 24, 19–33. 10.1039/cs9952400019

[B3] Buettner G. R. (1987). Spin trapping: esr parameters of spin adducts. Free Radic. Biol. Med. 3, 259–303. 10.1016/S0891-5849(87)80033-3 2826304

[B4] Chan W. Shiao N. Lu P. (2006). CdSe quantum dots induce apoptosis in human neuroblastoma cells via mitochondrial-dependent pathways and inhibition of survival signals. Toxicol. Lett. 167, 191–200. 10.1016/j.toxlet.2006.09.007 17049762

[B5] Cho S. J. Maysinger D. Jain M. Röder B. Hackbarth S. Winnik F. M. (2007). Long-Term exposure to CdTe quantum dots causes functional impairments in live cells. Langmuir 23, 1974–1980. 10.1021/la060093j 17279683

[B6] Choi H. S. Liu W. Misra P. Tanaka E. Zimmer J. P. Ipe B. I.. (2007). Renal clearance of quantum dots. Nat. Biotechnol. 25, 1165–1170. 10.1038/nbt1340 17891134 PMC2702539

[B7] Connor E. E. Mwamuka J. Gole A. Murphy C. J. Wyatt M. D. (2005). Gold nanoparticles are taken up by human cells but do not cause acute cytotoxicity. Small 1, 325–327. 10.1002/smll.200400093 17193451

[B8] Courtney C. M. Goodman S. M. McDaniel J. A. Madinger N. E. Chatterjee A. Nagpal P. (2016). Photoexcited quantum dots for killing multidrug-resistant bacteria. Nat. Mater. 15, 529–534. 10.1038/nmat4542 26779882

[B9] Courtney C. M. Goodman S. M. Nagy T. A. Levy M. Bhusal P. Madinger N. E.. (2017). Potentiating antibiotics in drug-resistant clinical isolates via stimuli-activated superoxide generation. Sci. Adv. 3:e1701776. 10.1126/sciadv.1701776 28983513 PMC5627983

[B10] Dai T. Huang Y.-Y. Hamblin M. R. (2010). Photodynamic therapy for localized infections – state of the art. Photodiagn. Photodyn. Ther. 6, 170–188. 10.1016/j.pdpdt.2009.10.008 19932449 PMC2811240

[B11] Dumas E. Gao C. Suffern D. Bradforth S. E. Dimitrijevic N. M. Nadeau J. L. (2010). Interfacial charge transfer between CdTe quantum dots and gram negative vs gram positive bacteria. Environ. Sci. Technol. 44, 1464–1470. 10.1021/es902898d 20085260

[B12] Feng Z. V. Gunsolus I. L. Qiu T. A. Hurley K. R. Nyberg L. H. Frew H.. (2015). Impacts of gold nanoparticle charge and ligand type on surface binding and toxicity to gram-negative and gram-positive bacteria. Chem. Sci. 6, 5186–5196. 10.1039/C5SC00792E 29449924 PMC5669217

[B13] Finkelstein E. Rosen G. M. Rauckman E. J. (1980). Spin trapping of superoxide and hydroxyl radical: practical aspects. Arch. Biochem. Biophys. 200, 1–16. 10.1016/0003-9861(80)90323-9 6244786

[B14] Gao L. Liu R. Gao F. Wang Y. Jiang X. Gao X. (2014). Plasmon-Mediated generation of reactive oxygen species from near-infrared light excited gold nanocages for photodynamic therapy *in vitro*. ACS Nano 8, 7260–7271. 10.1021/nn502325j 24992260

[B15] Gao X. Cui Y. Levenson R. M. Chung L. W. K. Nie S. (2004). *In vivo* cancer targeting and imaging with semiconductor quantum dots. Nat. Biotechnol. 22, 969–976. 10.1038/nbt994 15258594

[B16] Glozman A. Lifshitz E. Hoppe K. Rogach A. L. Welllr H. Echymüller A. (2001). Optically detected magnetic resonance of thiol-capped CdTe nanocrystals. Isr. J. Chem. 41, 39–44. 10.1560/GVFT-60DT-8Y0E-7DR9

[B17] Gomes S. A. O. Vieira C. S. Almeida D. B. Santos-Mallet J. R. Menna-Barreto R. F. S. Cesar C. L.. (2011). CdTe and CdSe quantum dots cytotoxicity: a comparative study on microorganisms. Sensors 11, 11664–11678. 10.3390/s111211664 22247686 PMC3252003

[B18] Grabolle M. Spieles M. Lesnyak V. Gaponik N. Eychmüller A. Resch-Genger U. (2009). Determination of the fluorescence quantum yield of quantum dots: suitable procedures and achievable uncertainties. Anal. Chem. 81, 6285–6294. 10.1021/ac900308v

[B19] Hamblin M. R. Hasan T. (2014). Photodymamic therapy: a new antimicrobial approach to infectious disease? Photochem. Photobiol. Sci. 3, 436–450. 10.1039/b311900a 15122361 PMC3071049

[B20] World Health Organization (2018). WHO Antimicrobial Resistance. Available Online at: http://www.who.int/mediacentre/factsheets/fs194/en/

[B21] Hirsch L. R. Stafford R. J. Bankson J. A. Sershen S. R. Rivera B. Price R. E.. (2003). Nanoshell-Mediated near-infrared thermal therapy of tumors under magnetic resonance guidance. Proc. Natl. Acad. Sci. U.S.A. 100, 13549–13554. 10.1073/pnas.2232479100 14597719 PMC263851

[B22] Huang X. El-Sayed I. H. Qian W. El-Sayed M. A. (2006). Cancer cell imaging and photothermal therapy in the near-infrared region by using gold nanorods. J. Am. Chem. Soc. 128, 2115–2120. 10.1021/ja057254a 16464114

[B23] Imlay J. A. (2013). The molecular mechanisms and physiological consequences of oxidative stress: lessons from a model bacterium. Nat. Rev. Microbiol. 11, 443–454. 10.1038/nrmicro3032 23712352 PMC4018742

[B24] Ipe B. I. Lehnig M. Niemeyer C. M. (2005). On the generation of free radical species from quantum dots. Small 1, 706–709. 10.1002/smll.200500105 17193510

[B25] Jarvi M. T. Niedre M. J. Patterson M. S. Wilson B. C. (2006). Singlet Oxygen Luminescence Dosimetry ( SOLD ) for photodynamic therapy: current status, challenges and future prospects. Photochem. Photobiol. 82, 1198–1210. 10.1562/2006-05-03-IR-891 16808593

[B26] Katari J. E. B. Colvin V. L. Alivisatos A. P. (1994). X-ray photoelectron spectroscopy of CdSe nanocrystals with applications to studies of the nanocrystal surface. J. Phys. Chem. 98, 4109–4117. 10.1021/j100066a034

[B27] Kirchner C. Liedl T. Kudera S. Pellegrino T. Javier A. M. Gaub H. E.. (2005). Cytotoxicity of colloidal CdSe and CdSe/ZnS nanoparticles. Nano Lett. 5, 331–338. 10.1021/nl047996m 15794621

[B28] Lovrić J. Bazzi H. S. Cuie Y. Fortin G. R. A. Winnik F. M. Maysinger D. (2005). Differences in subcellular distribution and toxicity of green and red emitting CdTe quantum dots. J. Mol. Med. 83, 377–385. 10.1007/s00109-004-0629-x 15688234

[B29] Lu Z. Li C. M. Bao H. Qiao Y. Toh Y. Yang X. (2008). Mechanism of antimicrobial activity of CdTe quantum dots. Langmuir 24, 5445–5452. 10.1021/la704075r 18419147

[B30] Ma J. Chen J. Y. Idowu M. Nyokong T. (2008). Generation of singlet oxygen via the composites of water-soluble thiol-capped CdTe quantum dots-sulfonated aluminum phthalocyanines. J. Phys. Chem. B 112, 4465–4469. 10.1021/jp711537j 18363400

[B31] Medintz I. L. Uyeda H. T. Goldman E. R. Mattoussi H. (2005). Quantum dot bioconjugates for imaging, labelling and sensing. Nat. Mater. 4, 435–446. 10.1038/nmat1390 15928695

[B32] Michalet X. Pinaud F. F. Bentolil L. A. Tsay J. M. Doose S. Li J. J.. (2005). Quantum dots for live cells, *in vivo* imaging and diagnostics. Science 307, 538–545. 10.1126/science.1104274 15681376 PMC1201471

[B33] Nagy A. Steinbrück A. Gao J. Doggett N. Hollingsworth J. A. Iyer R. (2012). Comprehensive analysis of the effects of cdse quantum dot size, surface charge, and functionalization on primary human lung cells. ACS Nano 6, 4748–4762. 10.1021/nn204886b 22587339

[B34] Pitout J. D. D. Laupland K. B. (2008). Extended-Spectrum β-Lactamase-Producing Enterobacteriaceae: an emergin public-health concern. Lancet 8, 159–166. 10.1016/S1473-3099(08)70041-0 18291338

[B35] Reynolds T. S. Courtney C. M. Erickson K. E. Wolfe L. M. Chatterjee A. Nagpal P.. (2017). ROS mediated selection for increased NADPH availability in *Escherichia Coli*. Biotechnol. Bioeng. 114, 2685–2689. 10.1002/bit.26385 28710857

[B36] Schneider R. Wolpert C. Guilloteau H. Balan L. Lambert J. Merlin C. (2009). The exposure of bacteria to CdTe-core quantum dots: the importance of surface chemistry on cytotoxicity. Nanotechnology 20:225101. 10.1088/0957-4484/20/22/225101 19433881

[B37] Thannickal V. J. Fanburg B. L. (2000). Reactive oxygen species in cell signaling. Am. J. Physiol. Lung Cell. Mol. Physiol. 279, L1005–L1028. 10.1152/ajplung.2000.279.6.L1005 11076791

[B38] Trachootham D. Alexandre J. P H. (2009). Targeting cancer cells by ROS-mediated mechanisms: a radical therapeutic approach? Nat. Rev. Drug Discov. 8, 579–591. 10.1038/nrd2803 19478820

[B39] Tsay J. M. Pflughoefft M. Bentolila L. A. Weiss S. (2004). Hybrid approach to the synthesis of highly luminescent CdTe/ZnS and CdHgTe/ZnS nanocrystals. J. Am. Chem. Soc. 126, 1926–1927. 10.1021/ja039227v 14971912

[B40] Valko M. Leibfritz D. Moncol J. Cronin M. T. D. Mazur M. Telser J. (2007). Free radicals and antioxidants in normal physiological functions and human disease. Int. J. Biochem. Cell Biol. 39, 44–84. 10.1016/j.biocel.2006.07.001 16978905

[B41] Wondrak G. T. (2009). Redox-Directed cancer therapeutics: molecular mechanisms and opportunities. Antioxid. Redox Signal. 11, 3013–3069. 10.1089/ars.2009.2541 19496700 PMC2824519

[B42] Yu B. P. (1994). Cellular defenses against damage from reactive oxygen species. Physiol. Rev. 74, 139–162. 10.1152/physrev.1994.74.1.139 8295932

[B43] Yu W. W. Qu L. Guo W. Peng X. (2003). Experimental determination of the extinction coefficient of CdTe, CdSe, and CdS nanocrystals. Chem. Mater. 15, 2854–2860. 10.1021/cm034081k

[B44] Zeng C. Ramos-Ruiz A. Field J. A. Sierra-Alvarez R. (2015). Cadmium Telluride (CdTe) and Cadmium Selenide (CdSe) leaching behavior and surface chemistry in response to pH and O2. J. Environ. Manage. 154, 78–85. 10.1016/j.jenvman.2015.02.033 25710599

[B45] Zwietering M. H. Jongenburger I. Rombouts F. M. Vant Riet K. (1990). Modeling of the bacterial growth curve. Appl. Environ. Microbiol. 56, 1875–1881. 16348228 10.1128/aem.56.6.1875-1881.1990 PMC184525

