# Supplementary material for: Designing Superoxide-Generating Quantum Dots for Selective Light-Activated Nanotherapy
Source: Front Chem. 2018 Mar 14;6:46. doi: 10.3389/fchem.2018.00046 (PMC5861142; doi:10.3389/fchem.2018.00046)
Supplement: Supplementary file 1 [file DataSheet1.DOCX]

Designing Superoxide-Generating Quantum Dots for Selective Light-Activated Nanotherapy

Samuel M. Goodman,^1,2,#^ Max Levy,^1,2,#^ Fei-Fei Li,^1,2^ Yuchen Ding,^2,3^ Colleen M. Courtney,^1^ Partha P. Chowdhury,^1,2^ Annette Erbse,^3^ Anushree Chatterjee,^1^ Prashant Nagpal^1,2,4*^

*^1^ Chemical and Biological Engineering, University of Colorado Boulder, Boulder, CO 80303*

*^2^ Renewable and Sustainable Energy Institute, University of Colorado Boulder, Boulder, CO 80303*

*^3^ Chemistry and Biochemistry, University of Colorado Boulder, Boulder, CO 80303*

*^4^ Materials Science and Engineering, University of Colorado Boulder, Boulder, CO 80303*

*^#^ These authors contributed equally to this work*

**Corresponding Author. Email:* [*pnagpal@colorado.edu*](mailto:pnagpal@colorado.edu)

**Electronic Supplementary Information (ESI)**


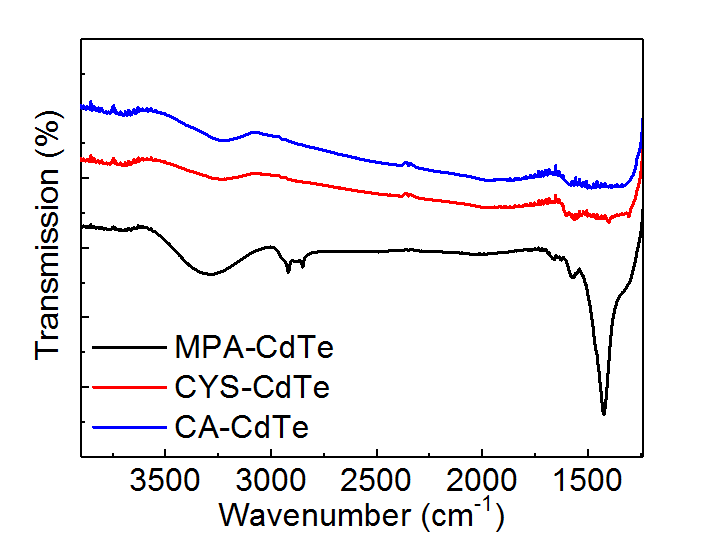


**Figure S1** – Fourier-transform infrared spectroscopy measurements of CdTe with different organic ligands. MPA- and Cys-CdTe prepared as described in methods. CA-CdTe also prepared as described—with ligand exchange method starting from MPA-CdTe. Peak near 1500 cm^-1^ indicative of MPA attachment to QD surface. Broad peaks near 3200 cm^-1^ (and absence of MPA peaks) indicate appropriate cysteine and cysteamine attachment.


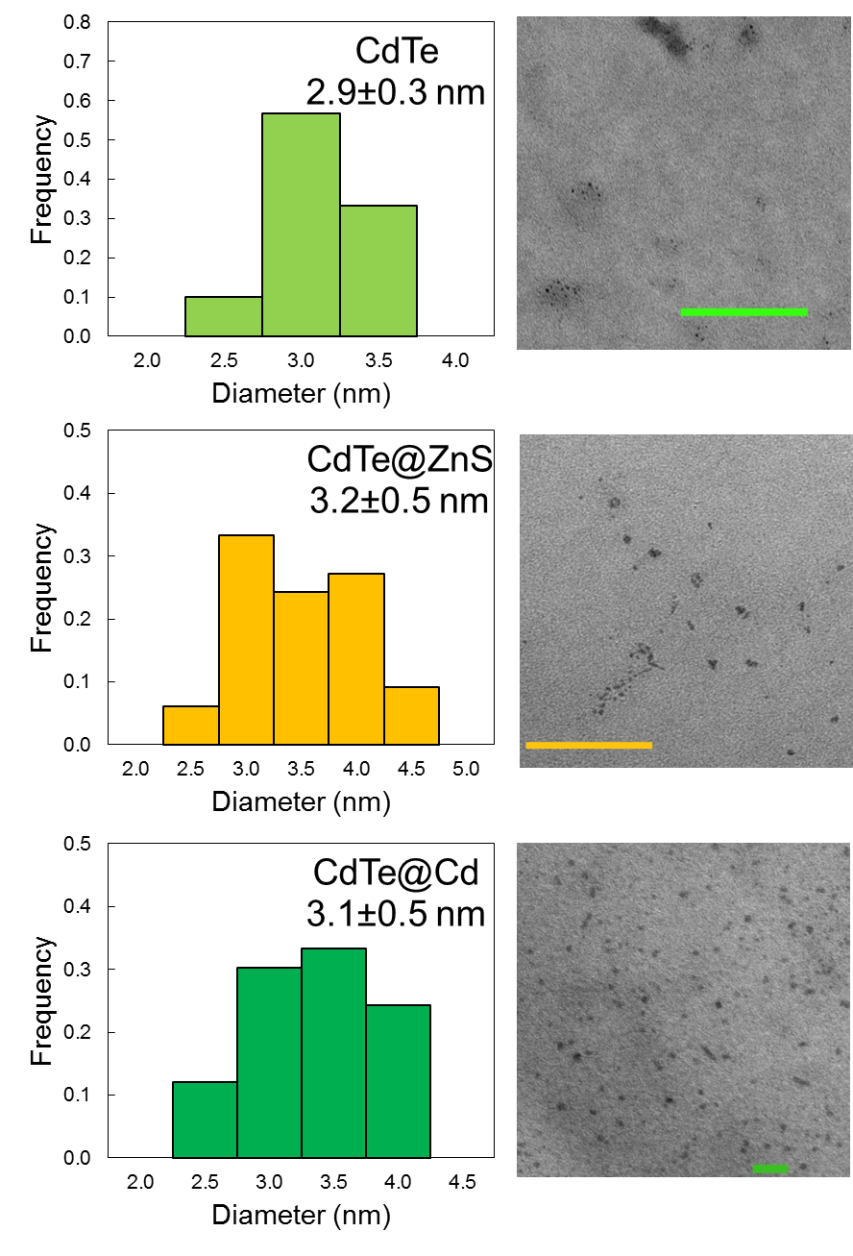


**Figure S2** – TEM images and size distribution histograms of the core and core-shell particles. Distributions and average diameters are the result of the analysis of 30 particles. Scale bars are 100 nm for CdTe and CdTe@ZnS and 20 nm for CdTe@Cd.


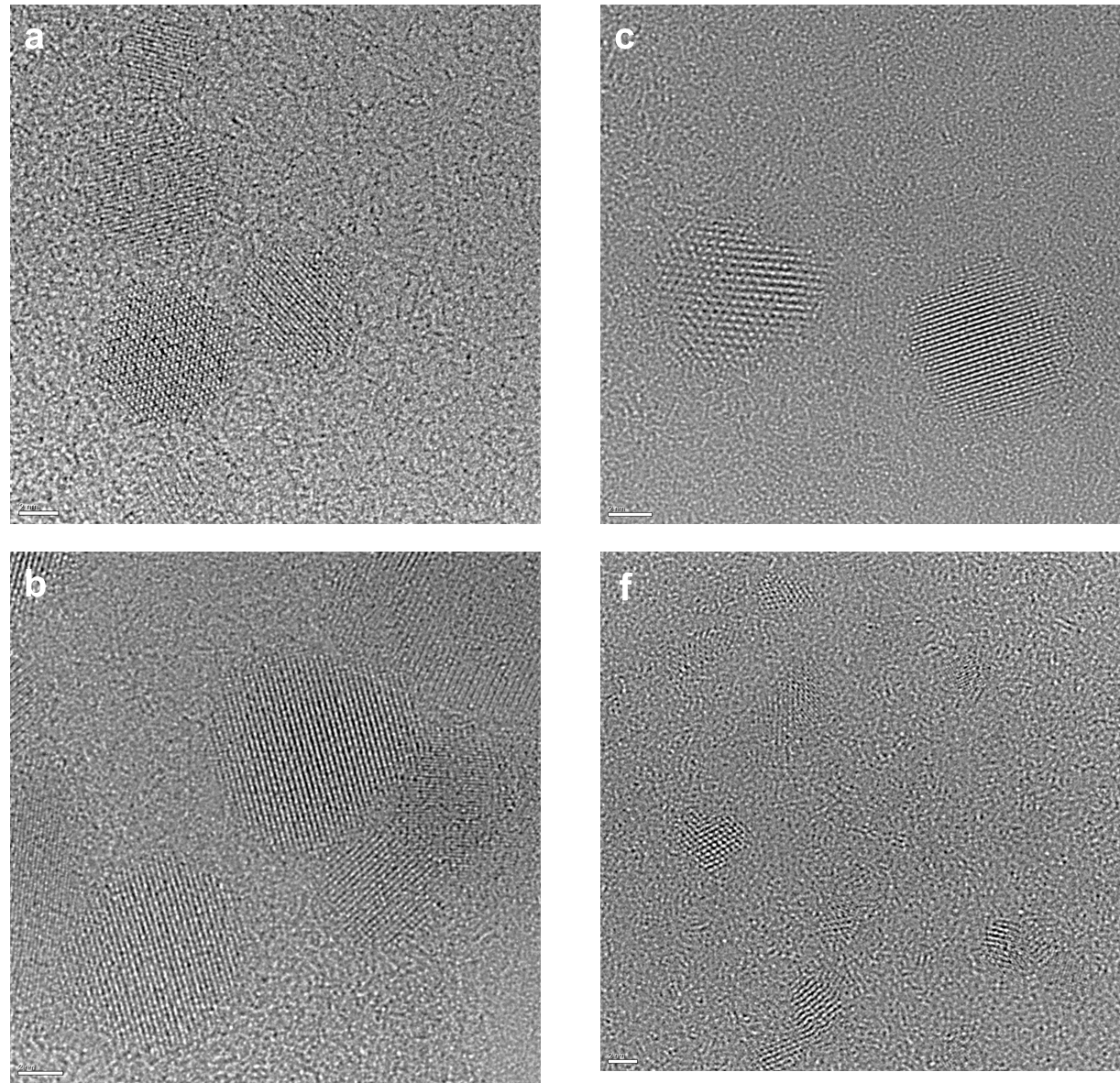


**Figure S3** – HRTEM images of CdTe. Scale bar: 2nm


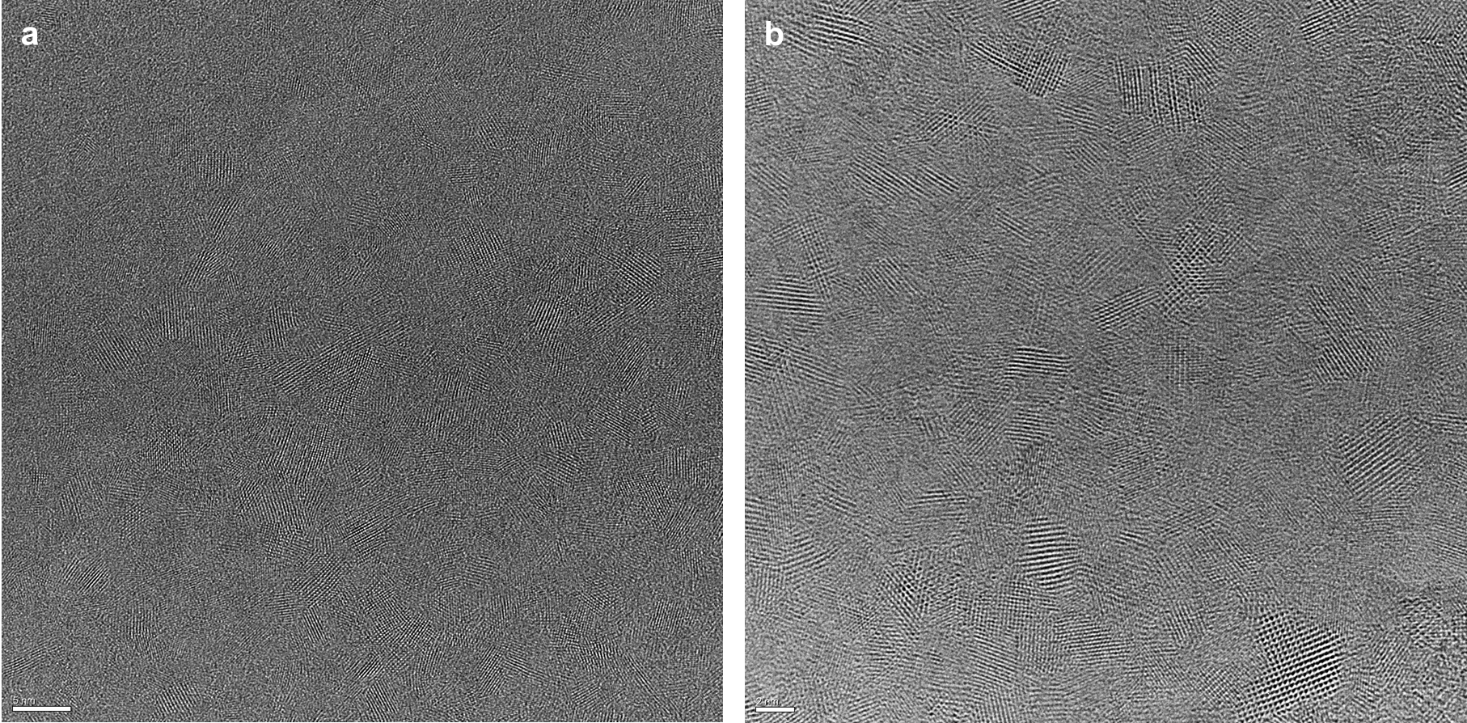


**Figure S4** – HRTEM images of CdTe. a) Scale bar: 5 nm; b) scale bar: 2 nm


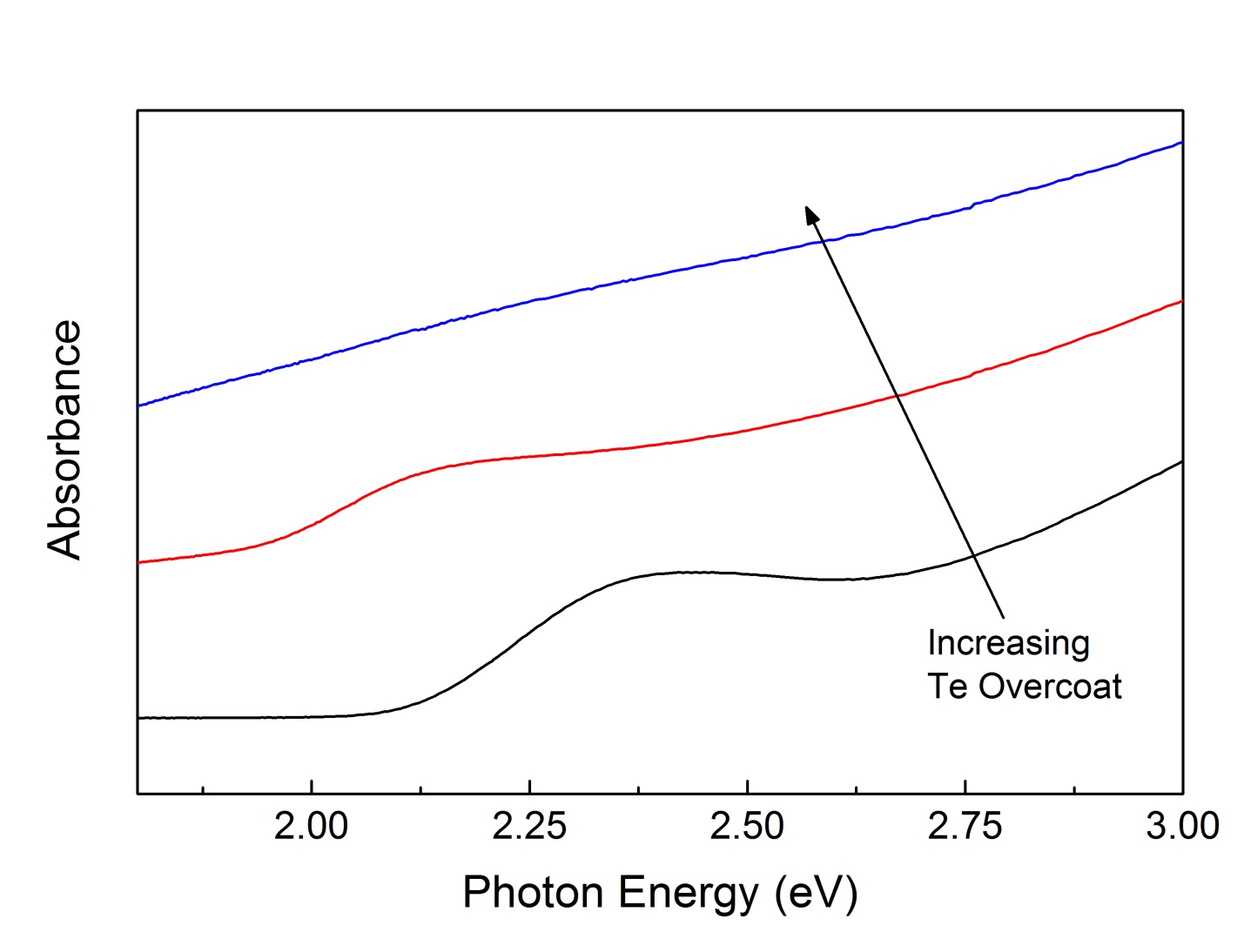


**Figure S5** – Absorbance spectra showing the effect of adding an analogous Te overcoat (CdTe@Te) on the optical properties of the nanoparticles. Compared to the cores (black line) adding additional tellurium causes a large red-shift in the peak position and causes the background to assume scattering features (red line). Adding the same molar equivalent Te as used in the CdTe@Cd core-shells results in a spectrum devoid of excitionic features which is dominated by scattering extinction (blue line).
